# Mini Tracheostomy for Obstructive Sleep Apnea: An Evidence Based Proposal

**DOI:** 10.1155/2016/7195349

**Published:** 2016-01-26

**Authors:** Macario Camacho, Soroush Zaghi, Edward T. Chang, Sungjin A. Song, Blake Szelestey, Victor Certal

**Affiliations:** ^1^Otolaryngology-Head and Neck Surgery, Tripler Army Medical Center, HI 96859, USA; ^2^Stanford Hospital and Clinics, Department of Psychiatry and Behavioral Sciences, Division of Sleep Medicine, Stanford, CA 94063, USA; ^3^Department of Head and Neck Surgery, David Geffen School of Medicine at UCLA, Los Angeles, CA 90095, USA; ^4^Department of Otolaryngology-Head and Neck Surgery, Division of Sleep Surgery and Medicine, Stanford, CA 94063, USA; ^5^Wright State University Boonshoft School of Medicine, Dayton, OH 45435, USA; ^6^Department of Otorhinolaryngology, Sleep Medicine Centre, Hospital CUF Porto, 4100-180 Porto, Portugal; ^7^Center for Research in Health Technologies and Information Systems (CINTESIS), University of Porto, 4200-450 Porto, Portugal

## Abstract

*Objective*. To search for articles evaluating the use of tracheostomies (either permanent stomas or tracheostomy tubes) in adult obstructive sleep apnea (OSA) patients and to evaluate the potential for the use of mini tracheostomies as treatment for OSA.* Study Design*. Systematic review.* Methods*. Nine databases were searched from inception through July 21, 2015.* Results*. The overall tracheostomy search yielded 516 articles, of which eighteen studies provided polysomnographic data. No study was identified (empty review) for the use of mini tracheostomies for treating OSA. The mini tracheostomy search yielded ninety-five articles which describe findings for either mini tracheostomy kits (inner cannula diameter of 4 mm) or the performance of mini tracheotomies. Six articles described the use of mini tracheostomies as a temporary procedure to relieve acute upper airway obstruction and none described the use for OSA. For tracheostomy stomal sites, suturing the skin directly to the tracheal rings with defatting can minimize stomal site collapse. The smallest tracheostomy stomal size that can successfully treat OSA has not been described.* Conclusion*. Mini tracheostomies as small as 4 mm have been successfully used in the short term to relieve upper airway obstruction. Given that polysomnography data are lacking, additional research is needed.

## 1. Introduction

Obstructive sleep apnea (OSA) is a common disorder that can cause repetitive nighttime upper airway obstructions with associated oxygen desaturations and/or arousals which often results in daytime hypersomnolence. With the advent of polysomnography, patients have the benefit of undergoing a formal attended overnight sleep study. This provides a diagnosis and categorization of either no, mild, moderate, or severe OSA [1]. There are several areas of narrowing in the upper airway including the nasal cavity (turbinates [2] and septum [3]), nasopharynx (adenoids), oropharynx, and hypopharynx [4]. Currently there are many medical [5–7] and surgical [8–11] treatment options for OSA with continuous positive airway pressure (CPAP) treatment being the gold standard therapy. Prior to the development of CPAP devices [12] in the 1980s, tracheotomies were performed as the standard of care for treating OSA patients, especially severe OSA patients with significant comorbidities [13].

The success of treating OSA patients with a tracheostomy led to several patients being referred to surgical departments from sleep medicine clinics. At Stanford, Drs. Powell, Riley, and Guilleminault have provided recommendations as to when to perform tracheostomies, including (1) emergent need to establish and ensure a patent airway, (2) geographic location where there is a lack of specialized equipment or surgical expertise to offer an alternative procedure, (3) selecting patients with morbid obesity (BMI, 40 kg/m^2^), severe hypoxemia (SaO_2_ ≤ 70%), serious arrhythmia, or dysrhythmia, and (4) the patient being refractory to medical management with CPAP and/or other treatment modalities [14].

Recent meta-analyses have demonstrated that tracheostomies are effective for treating OSA [15], even in morbidly obese patients [16]. Both tubed and tubeless (permanent) tracheostomies are used as treatment for OSA [17]. By definition, OSA occurs during sleep; therefore, while awake, OSA patients with tracheostomies sometimes either choose to place a speaking valve over the tube or temporarily remove the tube and cover the stoma with concealing adhesive covers or jewelry. Since the tracheostomy stomal site required to accommodate the tracheostomy tube is generally quite large (>1.2 cm), the size can be a deterrent for patients to receive a tracheostomy. One method to make tracheostomies more appealing to patients may be to decrease the size of the tracheostomy stoma, thereby allowing for easier concealment and easier coverage of the stoma during speaking. The minimum size needed for tracheostomy stomal sites in order to effectively treat OSA is currently unknown.

Mini tracheostomies have been described as early as 1984 as a method for efficient suctioning of pulmonary secretions [18]. The use of mini tracheostomies has also been reported for emergency airway situations, for temporary use of MTK tubes after surgery, and for both cricothyroidotomy and emergency tracheostomy training purposes. Mini tracheostomies were initially placed through the cricothyroid membrane using a guarded knife and introducer initially [19]. Over the years, authors such as van Heurn et al., who have significant experience with mini tracheostomies, have placed them in the subcricoid position [19]. The objective of this study was to search the international literature for articles evaluating the use of tracheostomies (either tubed or permanent stomas) in adult obstructive sleep apnea patients and to evaluate the potential for the use of mini tracheostomies as treatment for OSA.

## 2. Methods

Authors Macario Camacho, Soroush Zaghi, and Victor Certal searched Embase, Google Scholar, PubMed, Scopus, Book Citation Index-Science (since 2005), Cumulative Index to Nursing and Allied Health, Conference Proceedings Citation Index-Science (since 1990), The Cochrane Library, and Web of Science independently through July 21, 2015. Two main searches were performed: the first search was an “overall tracheostomy” search in order to identify studies that provided polysomnographic data before and after tracheostomy. The second search was to identify studies in which mini tracheostomies were used specifically for either treating acute airway obstruction or treating OSA. An example of a search on PubMed was (“Sleep Apnea Sydromes” [MeSH]” AND “Tracheostomy” [MeSH]). An example of the mini tracheostomy search was (((“little” OR “mini^*∗*^” OR “small” OR “tiny”) AND “trach^*∗*^”) OR “minitracheostomy”).

The Preferred Reporting Items for Systematic Reviews and Meta-Analysis (PRISMA) statement was downloaded and adhered to as much as possible [20].

### 2.1. Study Selection

Study inclusion criteria are as follows: (1) patients: adults with OSA, (2) intervention: tracheostomy or mini tracheostomy, (3) comparison: qualitative data or quantitative polysomnographic, sleepiness, or quality of life data before and after mini tracheostomy, (4) outcome: qualitative outcomes, polysomnography variables, or complications, and (5) study design: all including case reports, case series, cohort studies, and randomized or randomized-controlled trials. Exclusion criteria are as follows: (1) studies on children or (2) studies for patients with central sleep apnea.

## 3. Results

### 3.1. Tracheostomy for OSA

**Figure 1:**
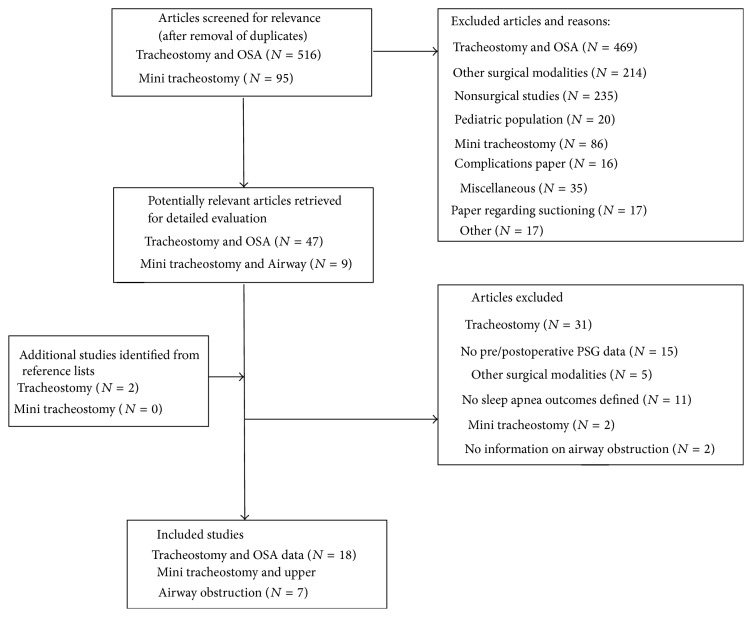
Flow diagram for tracheostomy studies and mini tracheotomy studies. *N*: number of articles.

The overall tracheostomy and sleep search yielded 516 articles. There were eighteen studies that reported obstructive sleep apnea polysomnography, sleepiness, or quality of life outcomes before and after tracheostomy [13, 21–37]; see Figure 1. None of the studies discussed mini tracheostomies as a subcategory for tracheostomy technique used for the treatment of OSA. A large percentage of the patients who use tracheostomies as treatment for OSA are obese or morbidly obese [16]. Because of the neck adiposity, many studies have demonstrated that obese OSA patients may have persistent obstruction despite having a tracheostomy tube in place [16].

### 3.2. Tubed versus Tubeless Tracheostomies

Tracheostomies with cuffed tubes are often utilized in the early perioperative period and these are switched to smaller cuffless tubes after the first tracheostomy tube exchange around postoperative days five to seven. It was recognized that the submental and neck adiposity could occlude the tracheostomy tube when the neck was flexed or if the patients turned their neck while asleep; thereby, in rare cases the apnea-hypopnea index did not change at all after surgery [21]. In order to overcome the issue of increased distance from the skin edge to the lumen of the trachea, there have been several tracheostomy tube designs that increase the tube length. These designs include the extended Shiley tracheostomy tubes, Vygon tracheostomy tubes, Portex extended length tubes, and the adjustable Proximal Extension Bivona tracheostomy tubes. Even with the extended length tubes, the patients may still occlude the tracheostomy site secondary to submental and neck adiposity. Other methods to decrease the problem of adiposity obstructing the tracheostomy tube include neck defatting techniques via direct excision of the fatty tissue or via suction lipectomy [17, 38].

### 3.3. Suction Lipectomy and Defatting Techniques

Several techniques have been published in the literature for performing a permanent tracheotomy. Valero and Alroy described the conversion of a standard tubed tracheostomy into a permanent tracheostomy in a patient with acquired micrognathia and excessive daytime hypersomnolence [39]. There are various techniques for performing permanent tracheotomy stomal sites, such as those described by Fee Jr. and Ward [17], Gross et al. [38], and others [40, 41]. In a morbidly obese OSA patient, Eliashar et al. [42] described a tube-free tracheostomy which is skin-lined, noncollapsing, nonstenosing and has a self-sustaining stoma that does not require a cannula to maintain the tract open. The technique used by Elisahar et al. is performed under general anesthesia: a horizontal, omega-shaped incision is made 1 cm above the level of the clavicles with a lipectomy of the suprasternal region in a subplatysmal plane; then the dissection proceeds to the medial third of tracheal rings 2 and 3 [42]. The skin flaps are thinned subcutaneously of excess adiposity and closed with vertical mattress sutures, creating a myocutaneous junction circumferentially around the stoma; a cuffed tracheostomy tube is placed for 12 hours; then it is removed and the stoma thereafter remains tube-free [42].

### 3.4. Tracheostomy Concealment

It is known that tracheostomies affect patients socially and psychologically. This has led to patients camouflaging the tracheostomy sites during the day with scarves, ascots, turtlenecks, and other clothing. In a letter to the editor, Boysen described a patient who had a custom fitted neck chain with the medallion covering the tracheostomy site [43].

### 3.5. Complications of Tracheostomy

Early and late complications can occur with tracheostomy tubes such as repeated obstruction secondary to neck adiposity occluding the tube, displacement or dislodgement of the tracheostomy tube, formation of granulation tissue, tracheal or tracheostomy tube mucous plugging, infection of the healing wound, pneumonia, recurrent bronchitis, and tracheoinnominate artery fistula formation [44]. Several studies have demonstrated a lower complication rate when permanent tracheostomies are created via either converting the traditional tracheotomy site with a tube to a permanent tracheostomy site or creation of a permanent tracheostomy as the initial surgery [17, 40]. There is a decreased amount of granulation tissue formation since the skin of the neck is brought down to the level of the trachea. The risk of tracheoinnominate fistula is nearly eliminated in the setting of having no tracheostomy tube pressing against the anterior tracheal wall. A few articles discuss complications which are similar to those seen in standard tracheostomies.

### 3.6. Tracheostomy Tube Sizes

Tracheostomy tubes come in many sizes. For obese adult patients, a Bivona hyperflexible, adjustable flange tracheostomy tube has an internal diameter of 8 millimeters (mm), an outer diameter of 11.7 mm, and a length of 130 mm. A single-cannula Shiley size 6 tracheostomy tube has an internal diameter of 6.4 mm, an outer diameter of 10.8 mm, and a length of 76 mm [45]. In contrast a neonatal tracheostomy tube has an internal diameter of 3.5 mm, an outer diameter of 5.22 mm, and a length of 32 mm.

### 3.7. Mini Tracheostomies

The mini tracheostomy search yielded ninety-five articles dating back to the mid-1980s which describe findings for mini tracheostomy kits (MTK) or the performance of mini tracheotomies. Of the ninety-five published studies, six [46–51] report the use in patients with acute upper airway obstructions, one [19] describes the use in two patients with upper airway obstructions (it did not specify if it was for an acute process), and none describes the use of mini tracheostomies for obstructive sleep apnea; see Figure 1. The MTK tracheostomy tubes are small-bore cannulas and have an internal diameter of 4 mm, an outer diameter of 5.4 mm, and a length of 90 mm. A large series by van Huern et al. in which they report findings for 50 patients who underwent mini tracheostomies noted a very low complication rate (10%) when performed in a subcricoid fashion percutaneously.

The majority were excluded from the study for the following reasons: description of late bleeding (*n* = one), granuloma formation (*n* = two), esophageal perforation (*n* = two), inhalation of a mini tracheostomy tube (*n* = one), use in laryngectomy patients (*n* = one), general complications (*n* = four), pneumothorax (*n* = three), described designing of a mini tracheostomy kit (*n* = one), prophylactic mini tracheostomy (*n* = three), sternal dehiscence (*n* = one), omentoplasty (*n* = one), ventilation study (*n* = three), suctioning (*n* = seventeen), training purposes (*n* = one), position of a tube verified with flexible endoscopy (*n* = one), retrograde intubation (*n* = one), review article (*n* = one), miscellaneous (thirty-five), techniques to decrease complications (*n* = one), operative technique paper (*n* = two), spinal cord injury (*n* = one), safety paper (*n* = one), and indications paper (*n* = two).

## 4. Discussion

There are five main points to this systematic review. First, tracheostomies can have high complication rates, especially when compared to CPAP. Given that CPAP is safe and the side effects are generally fairly benign, it should be attempted first. Potential complications from a tracheostomy include repeated obstruction secondary to neck adiposity occluding the tube, displacement or dislodgement of the tracheostomy tube, formation of granulation tissue, tracheal or tracheostomy tube mucous plugging, infection of the healing wound, pneumonia, recurrent bronchitis, and tracheoinnominate artery fistula formation [44]. By converting to a permanent tracheostomy, the number of complications decreased since the skin of the neck is brought down to the level of the trachea and there is no tube which can occlude or press against the trachea.

Second, patient selection is important. Patients need to understand the significance of having a tracheostomy and understand the associated lifestyle limitations (no swimming, etc.). Providers also should have a long discussion as to the differences between a tubed and a tubeless (permanent) tracheostomy. Although there are no strict criteria, generally the patient should (a) be refractory to medical management with CPAP and/or other treatment modalities, (b) be at a geographic location where there is a lack of specialized equipment or surgical expertise to offer an alternative procedure, and (c) be morbidly obese, have severe hypoxemia (SaO_2_ ≤ 70%), serious arrhythmia, or dysrhythmia [14].

Third, given that mini tracheostomies have been successfully used to bypass acute upper airway obstructions in the short term, we need to evaluate the potential use in the long term. The MTK tubes have an internal diameter of 4 mm; therefore, it could be hypothesized that 4 mm is the size that could also be used to treat adult OSA, compared to the single-cannula Shiley size 6 tracheostomy tube which has an internal diameter of 6.4 mm. Research is needed in order to evaluate the actual minimum size needed for adequate ventilation during sleep. Based on Poiseuille's Law, does the decreased flow rate of ventilation through the smaller diameter, longer tube cause too much of an increased force required to ventilation (e.g., a 40% flow rate through 4 mm compared to 6.4 mm internal diameter tube)? Limitations to creating mini tracheostomies include the risk of crusting and the difficulties associated with cleaning the tracheostomy tube and smaller tracheal stoma. It can be hypothesized that a mini tracheostomy would be easier to occlude completely with secretions and mucous or neck adiposity given that the size of the stoma is smaller. MTK tubes are made for adults; however, because other non-MTK tracheostomy tubes with smaller bore cannulas are typically used for children, the length of non-MTK tubes may be insufficient for morbidly obese adults; therefore, custom-made or extended length tracheostomies would need to be developed for use in adult OSA. Additionally, there may be a sex difference, since women have smaller tracheas than men, so their stoma could potentially be smaller than that created for men.

Fourth, whether a permanent mini tracheostomy stoma and tract would collapse is unknown. Typically, the application of negative pressure as encountered during inspiration leads to the application of Bernoulli's Principle about the flow of air through the tracheostomy stoma. Specifically, with negative pressure, the airway develops a streamlined, steady flow of air of constant density about an essentially frictionless surface [52]; see the following equation:

(1) $$\begin{array}{c} P+\frac{1}{2}\rho V^{2}+\rho gh=\text{constant}. \end{array}$$

Conditions for Bernoulli's Principle to apply are as follows: flow between 2 points lies on a streamline, the fluid has constant density, the flow is steady, and there is no friction [52]. *P* is pressure, *ρ* is density, *V* is velocity, *h* is elevation, and *g* is gravitational acceleration. These factors induce the necessary requirements for development of negative pressure about the flow of air through the stoma, thereby, increasing the likelihood of stomal collapse and/or occlusion from surrounding soft tissue. Direct suturing of the cartilaginous portions of the trachea to the overlying skin in a circumferential fashion, similar to a Bjork Flap (but with superior, inferior, and bilateral lateral skin-cartilaginous flaps), provides one method for decreasing the likelihood of stomal tract collapse. Eliashar et al. found that the stoma in permanent tracheostomies is noncollapsing, skin-lined, nonstenosing and is self-sustaining; however, the size was larger than 4 mm. Because airflow is faster in narrower tracts, there would be an increase in the negative pressure in the lumen of the tract and this could predispose to collapse in a noncartilaginous lined tract. A compromise that would help prevent or minimize collapse would be to have the patient use a mini tracheostomy tube at night only.

Fifth, patients could potentially be more accepting of mini tracheostomies compared to standard tubed or standard permanent tracheostomies given that they are easier to conceal or cover while awake. Mini tracheostomy stoma and tract that would accommodate a mini tracheostomy tube would likely need to be at least 6–8 mm in size given that the outer diameter of MTK tubes is 5.4 mm. Patients who want to keep the tube in all day may select a mini tracheostomy tube with a lower profile, and these may be more accepting for patients given that they are easier to conceal. Patients could also elect to keep the mini tracheostomy tube out of the stoma during the day and then replace it into the airway at night for sleeping purposes only. If a permanent stoma was to be created with suturing the skin directly to the tracheal rings, it has yet to be determined if a stomal diameter/tract size of 4 mm or smaller (3.5 mm, 3 mm, 2.5 mm, etc.) will be effective for treating OSA. Determining the effectiveness of mini tracheostomies and the minimum size adequate are both potential areas for future research.

*Limitations*. There are limitations. First, it is possible that despite our best efforts to identify mini tracheostomies in the literature, we may have missed finding a study with quantitative polysomnography data. However, multiple databases were systematically searched in an independent fashion. Second, this was an empty systematic review; however, given that tracheostomies remain important in the treatment of refractory OSA, the smallest size needed to overcome OSA remains important. Lastly, we were additionally limited in that no long-term studies were identified using mini tracheostomy.

## 5. Conclusion

Mini tracheostomies have been successfully used as small as 4 mm in the short term to relieve upper airway obstruction. Given that polysomnography data are lacking, additional studies are needed. Research can determine the very minimum tracheostomy size that is necessary (both with and without a mini tracheostomy tube in place) to provide the most effective airway with the lowest risk and complication profile.
